# Strain-Level Typing of *Streptococcus pyogenes* Using Optical DNA Mapping

**DOI:** 10.1021/acsinfecdis.5c00430

**Published:** 2025-10-15

**Authors:** Radhika N. Kunnath, Zahra Abbaspour, Anna Johnning, Karolin Frykholm, Marie Wrande, Albertas Dvirnas, Sriram KK, Christian G. Giske, Tobias Ambjörnsson, Linus Sandegren, Erik Kristiansson, Fredrik Westerlund

**Affiliations:** † Department of Life Sciences, 11248 Chalmers University of Technology, 412 96 Gothenburg, Sweden; ‡ Centre for Antibiotic Resistance Research in Gothenburg, CARe, P.O. Box 440, 405 30 Gothenburg, Sweden; § Department of Mathematical Sciences, Chalmers University of Technology and the University of Gothenburg, 412 96 Gothenburg, Sweden; ∥ Department of Systems and Data Analysis, 536563 Fraunhofer-Chalmers Centre, 412 88 Gothenburg, Sweden; ⊥ Department of Medical Biochemistry and Microbiology, Uppsala University, 751 23 Uppsala, Sweden; # Centre for Environmental and Climate Science, 5193 Lund University, Sölvegatan 12, 223 62 Lund, Sweden; ¶ Department of Laboratory Medicine, 27106 Karolinska Institutet, 141 52 Stockholm, Sweden; ∇ Department of Clinical Microbiology, Karolinska University Hospital, 171 76 Stockholm, Sweden; 9 Uppsala Antibiotic Center, Uppsala University, 751 23 Uppsala, Sweden

**Keywords:** bacterial typing, optical DNA mapping, *Streptococcus pyogenes*, genomics, nanofluidics, diagnostics

## Abstract

*Streptococcus pyogenes*, also known as group A *Streptococcus* (GAS), is a Gram-positive bacterial pathogen responsible for approximately 500,000 deaths globally per year. Therefore, surveillance of invasive GAS infections is of critical importance in understanding societal transmission. In this study, we propose an optical DNA mapping (ODM)-based assay for strain-level typing of *S. pyogenes*. Fluorescently labeled DNA molecules of lengths >150 kbp are stretched in nanochannels and imaged to reveal genomic information. A core genome alignment-based typing scheme is then utilized for identification at the strain level. Although our technique lacks the nucleotide-level resolution of whole-genome sequencing, it excels at detecting patterns across long stretches of DNA that remain inaccessible to short-read sequencing methods. Using clinical isolates, we demonstrate that the ODM assay can identify *S. pyogenes* at both the species level and at even higher taxonomic resolutions with high accuracy. Our typing scheme also shows a high correlation with standard *emm* subtyping for the analyzed samples. We conclude that ODM has the potential to deliver timely and cost-efficient strain-level bacterial identification.

## Graphical abstract

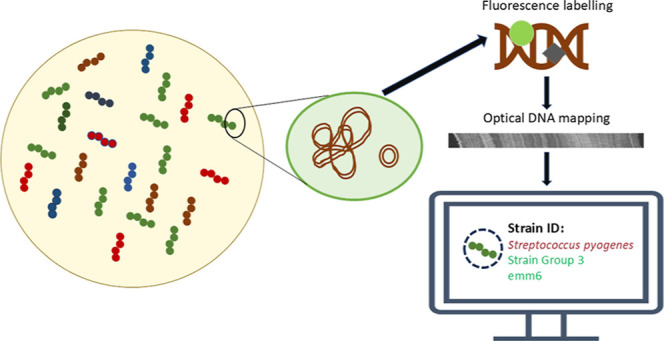

*Streptococcus pyogenes*, also known as group A *Streptococcus* (GAS), is a pathogenic Gram-positive bacterial species responsible for an estimated 500,000 deaths annually on a global scale.^1^ Millions of cases of GAS-related diseases are reported annually, primarily in the form of nonsevere infections, such as tonsillitis and impetigo. Nevertheless, severe infections can lead to life-threatening invasive GAS diseases^2^ and other complications, and in many countries, invasive GAS infections are notifiable diseases according to the communicable disease legislation. Furthermore, the incidence of GAS infections has been on the rise since the easing of the COVID-19 pandemic-related restrictions.^3^ Thus, the surveillance of GAS infections is of critical importance for public health.

For epidemiological reasons, it is important to accurately type the causative agent in a GAS infection to reveal transmission patterns of strains of public health concern. Currently, the standard epidemiological typing scheme for *S. pyogenes* is *emm* typing, which is based on the DNA sequence of a region of a single gene: 10–15% of the *emm* gene, which encodes the M protein, a major virulence factor of GAS.^4,5^ However, *emm* typing lacks sensitivity, and is often unable to determine virulence as strains with the same *emm* type may exhibit varying levels of pathogenicity.^6^ Multilocus sequence typing (MLST), which is based on the sequences of seven housekeeping genes, is also used to identify *S. pyogenes* isolates at the strain level. Studies have shown that there is a relationship between sequence types determined by MLST and *emm* type.^7,8^ However, both *emm* typing and MLST are based on the sequences of a few genes and, therefore, do not capture the full genetic diversity of *S. pyogenes* strains. Furthermore, the way these types are assigned to strains (typically using sequencing of PCR amplicons) has limited epidemiological and diagnostic relevance when dealing with mixed infections involving multiple strains.^9^ Moreover, these typing schemes do not provide any information about the evolutionary relationships between strains, which is vital for tracking how various strains evolve and adapt to selective pressures, such as exposure to antibiotics.^10^

We have previously demonstrated that competitive binding-based optical DNA mapping (ODM) can be used for typing of the Gram-negative bacteria *Escherichia coli* and *Klebsiella pneumoniae* at the strain level with high sensitivity and accuracy, even from uncultured clinical samples.^11,12^ Indeed, we demonstrated that ODM can accurately identify well-established *E. coli* lineages, such as ST131.^12^ The ODM assay visualizes sequence information along single DNA molecules, at least 150 kbp in length, stretched in nanofluidic channels.^13,14^ Labeling the DNA molecules with a non-fluorescent molecule that binds sequence-specifically (netropsin) and a fluorescent dye (YOYO-1), yields a fluorescence emission intensity variation along the DNA that reflects the underlying sequence.^15^ The intensity profile is then matched to a reference database of in silico-generated intensity profiles, which have been predicted from chromosomal DNA sequences. The method is as fast or faster than DNA sequencing, with a current sample-to-result turnaround time of 7 h.^12^ However, unlike sequencing-based assays, the ODM assay does not involve DNA amplification or require primer design. As it is a single-molecule technique, the assay has the potential to detect and identify all the bacteria from complex and mixed cultures. Indeed, we have previously demonstrated that both *E. coli* and *K. pneumoniae* could be correctly typed using ODM, both in mixes with four different strains and directly from uncultured urine samples, suggesting that the method has potential for clinical applications.^12^

In this study, we explored the potential of utilizing ODM based on competitive binding for typing *S. pyogenes* isolates at both the species and strain levels. In total, 11 clinical *S. pyogenes* strains isolated from blood and throat secretion samples, covering a broad diversity within the species, were analyzed with a true positive rate close to 100%. We also showed significant agreement between our typing approach, which is based on the evolutionary relationship between strains inferred from their core genomes, and the conventional *emm* typing scheme.

## Results and Discussion

We have evaluated the feasibility of using a competitive binding-based ODM typing assay^11,12^ for identifying and characterizing *S. pyogenes* at the strain level (Figure 1). Briefly, long DNA fragments (>150 kbp) are extracted from bacterial samples encased in agarose gel plugs, and DNA is stained according to a competitive binding-based protocol using the fluorescent dye YOYO-1 and the nonfluorescent, AT-specific molecule netropsin. The fluorescently stained DNA is stretched to nearly its full contour length in a nanofluidic device, visualized by fluorescence microscopy, and the emission intensity variation along the DNA, reflecting the local AT/GC ratio, is recorded, resulting in an experimental intensity profile.^15,16^ The experimental intensity profile of each DNA molecule is compared to theoretical profiles in a reference database derived from complete chromosomal sequences in the NCBI RefSeq database (35,316 chromosomal sequences, comprising 5488 bacterial species). Each combination of experimental and theoretical profiles generates a matching score based on the Pearson correlation coefficient (PCC; Supporting Information, Figure S2). In the reference database, each theoretical profile is associated with a species. The *S. pyogenes* references in the database are also associated with a strain group (SG) at two different strain-levels of taxonomic resolutions, and their *emm* type/cluster/pattern (Supporting Information, Table S1). We define the SGs as clades in a core genome-based phylogenetic tree of all *S. pyogenes* references, according to the branch lengths from the root (Supporting Information, Figure S1): one typing scheme based on a longer branch length creating fewer and larger SGs with more in-group diversity (the SG_Low_ scheme, all *S. pyogenes* reference genomes divided into three SGs) and one based on a shorter branch length creating more and smaller SGs with lower in-group diversity (the SG_High_ scheme, 40 SGs). For each experimental profile, only the high-confidence matches (the highest-ranking matches that fall within a predefined threshold) against the reference database is used for the remaining analysis (see Methods for details). If all the high-confidence matches corresponded to database references belonging to the same taxonomic group—e.g. a single species or a single SG—the experimental profile is deemed to match discriminatively at that taxonomic resolution and is reported in the typing results. Experimental profiles with high-confidence matches to multiple taxonomic groups are discarded. This ensures that only high-quality, unambiguous data is used in the typing result.

**Figure 1:**
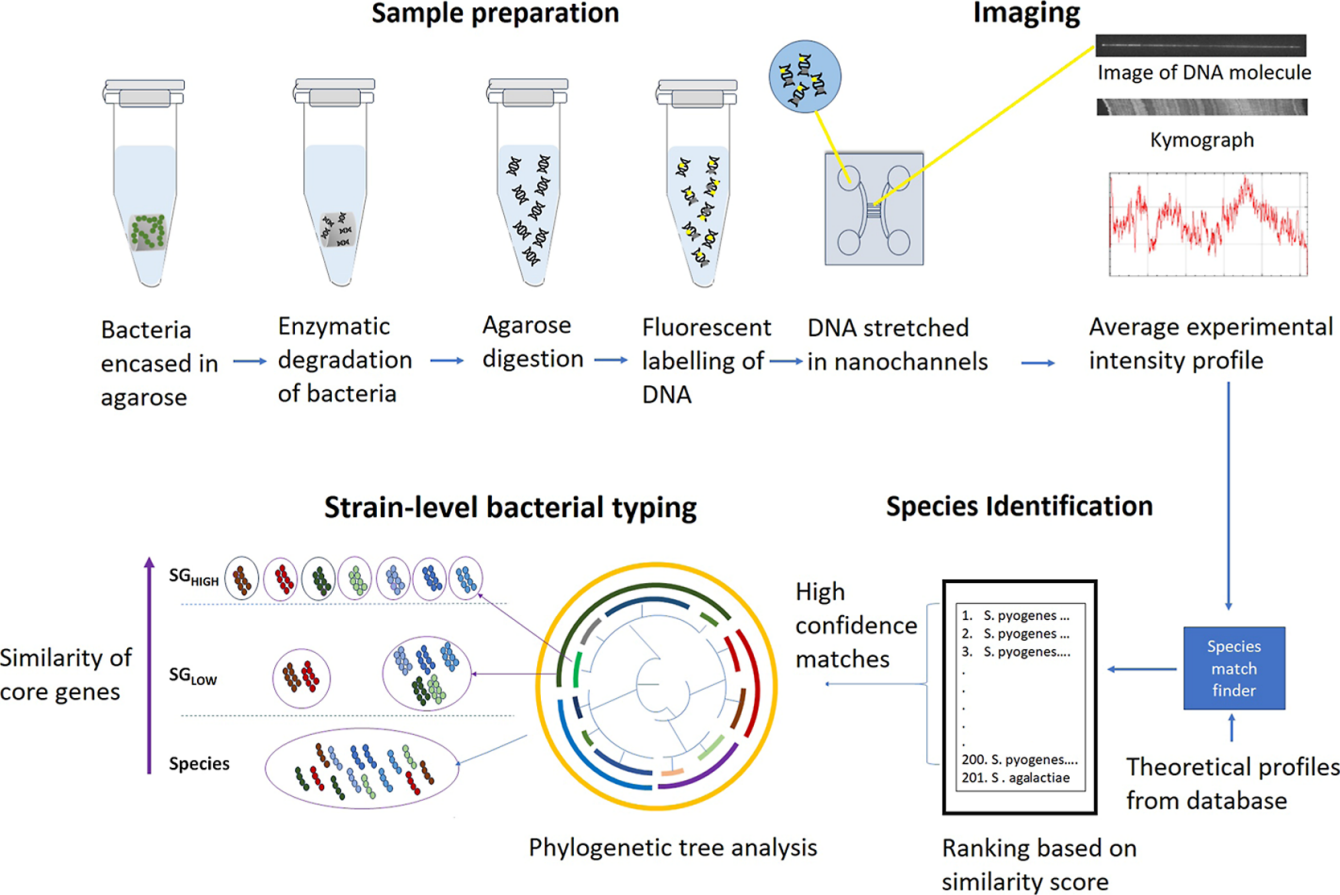
Schematic of the ODM assay showing the sample preparation, imaging, and data analysis steps. To extract ultrahigh molecular weight DNA, bacteria are first encased in an agarose gel plug and subjected to enzymatic treatments to degrade and digest the cells to leave only the DNA. The agarose is digested using agarase to release the DNA into the solution. Following this, the DNA is labeled using competitive binding of YOYO-1 and netropsin to the DNA. The labeled DNA molecules are stretched in nanochannels and imaged. An average intensity profile along the molecule is extracted from the movies and compared against theoretical intensity profiles from a database. For each intensity profile, the matches are ranked based on the Pearson correlation coefficient, and the highest-ranking matches (falling within a stringency parameter) are called high-confidence matches. A phylogenetic tree analysis based on a core-genome alignment is used to group the *S. pyogenes* reference strains into strain groups. If all the high-confidence matches match a single taxonomic group (species/strain group), the intensity profile is said to match discriminatively at that taxonomic resolution.

A total of 11 clinical *S. pyogenes* isolates, representing different SGs and *emm* types, were selected for the evaluation of the proposed method (Table 1). Two strains were isolated from clinical throat swabs (sample IDs PT1–PT2), and nine strains were isolated from blood samples (PB1–PB9). In total, 1271 experimental profiles were generated with an average of 115.5 profiles per isolate. To evaluate the impact of the parameter settings in the data analysis, we analyzed all experimental profiles while varying the matching stringency to the reference database. The stringency (parameter *C*_Diff_) determines the high-confidence matches: the smaller the C_Diff_is, the fewer reference matches are retained among the high-confidence matches. The stringency, therefore, affects both the number of discriminative profiles and the true positive rate (TPR). The analysis showed that a higher stringency resulted in a higher TPR, but fewer discriminative profiles to base the typing results on (Figure 2A,B). The effect of the stringency and the maximum length rescaling factor on the performance of the method was also evaluated (Figure S3). As in our previous studies,^11,12^ we concluded that a value of *C*_Diff_ = 0.05 was a good compromise between obtaining a high TPR for typing *S. pyogenes* while still retaining a sufficient number of discriminative profiles. This level of stringency resulted in an average TPR of 99.2% at the species level, 98.5% at the lower strain-level resolution (SG_Low_), and 93.2% at the higher strain-level resolution (SG_High_). The average proportion of discriminative profiles was 31.6%, 29.7%, and 13.7% for the species, the lower, and the higher strain-level resolutions, respectively.

**Table 1:** Analyzed Clinical *Streptococcus pyogenes* Isolates

Sample ID	SG_Low_	SG_High_	*emm* pattern	*emm* cluster	*emm* type
PT1	1	19	E	E4	28
PT2	1	19	E	E4	28
PB1	1	3	E	E4	2
PB2	1	38	E	E4	89
PB3	2	29	A–C	A–C5	3
PB4	1	20	E	E3	82
PB5	2	23	D	clade Y	95
PB6	2	29	A–C	A–C5	3
PB7	1	19	E	E4	28
PB8	1	13	A–C	A–C3	1
PB9	1	13	A–C	A–C3	1

**Figure 2:**
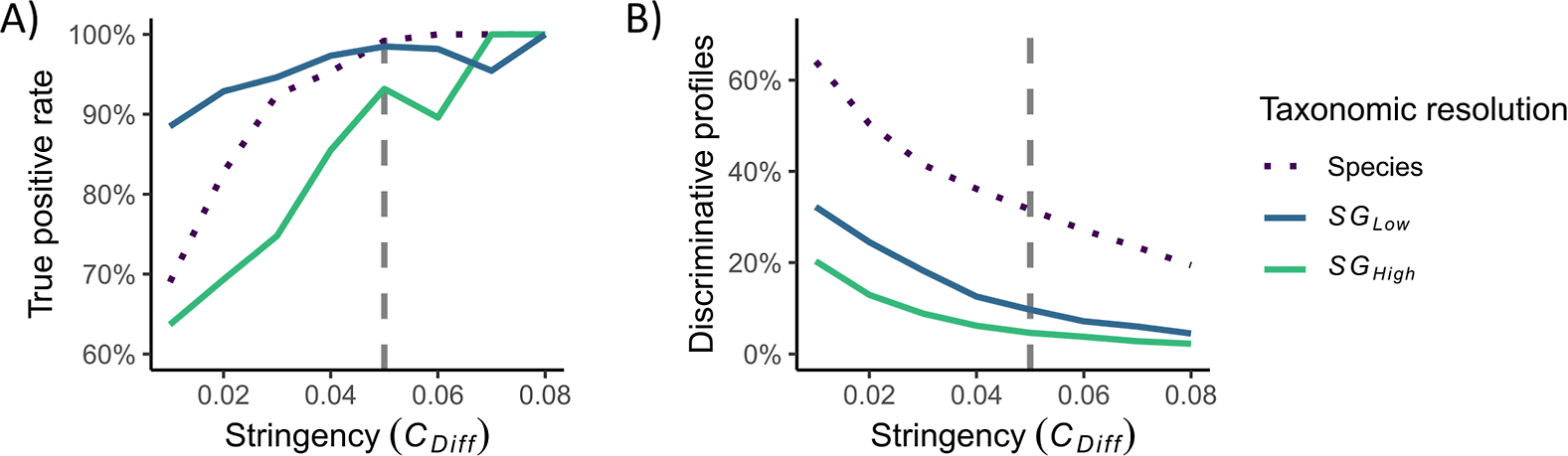
Effect of the matching stringency (parameter *C*_Diff_) on the typing results at the two selected strain levels (SG_Low_: blue; SG_High_: green) and the species level (dotted black). (A) The true positive rate, averaged over all 11 isolates, increases with increasing matching stringency for all taxonomic resolutions. (B) The proportion of discriminative profiles, averaged over all 11 isolates, decreases with increasing matching stringency for all taxonomic resolutions. The selected parameter setting (*C*_diff_ = 0.05) is marked with a vertical line (dashed gray) in both plots.

The competitive binding-based ODM assay could detect *S. pyogenes* at the species level with a TPR of 100% for all but two isolates (Figure 3). For these two isolates (PB1 and PB4), a total of three experimental profiles (0.23% of the total experimental profiles imaged in this study) matched discriminatively to the wrong species: *Sphingobacterium daejeonense*, *Yersinia ruckeri*, and *Methylomusa anaerophila*, respectively. These species are all distantly related to *S. pyogenes*, and the matches were, therefore, easily identifiable as false positives. We have previously recommended that a given sample should have at least three discriminately matching experimental profiles of the same species to ensure reliable results.^11^ The number of profiles that matched discriminatively at the species level was in the range of 18–59 (21–32% of the profiles in a sample). For the two strain-level resolutions, at SG_Low_ a TPR of 100% was achieved for ten isolates (83% for sample PB5) and at SG_High_, a TPR of 100% was achieved for nine isolates (50% for PB2 and 75% for PB4). At the lower strain-level resolution, the number of profiles that matched discriminatively was in the range of 5–11 (3–23% of the profiles in a sample), and for the higher strain-level resolution, it was in the range of 1–17 (1–15%). This shows that ODM can identify *S. pyogenes* at the species level and type it at the two selected strain levels with high accuracy.

**Figure 3:**
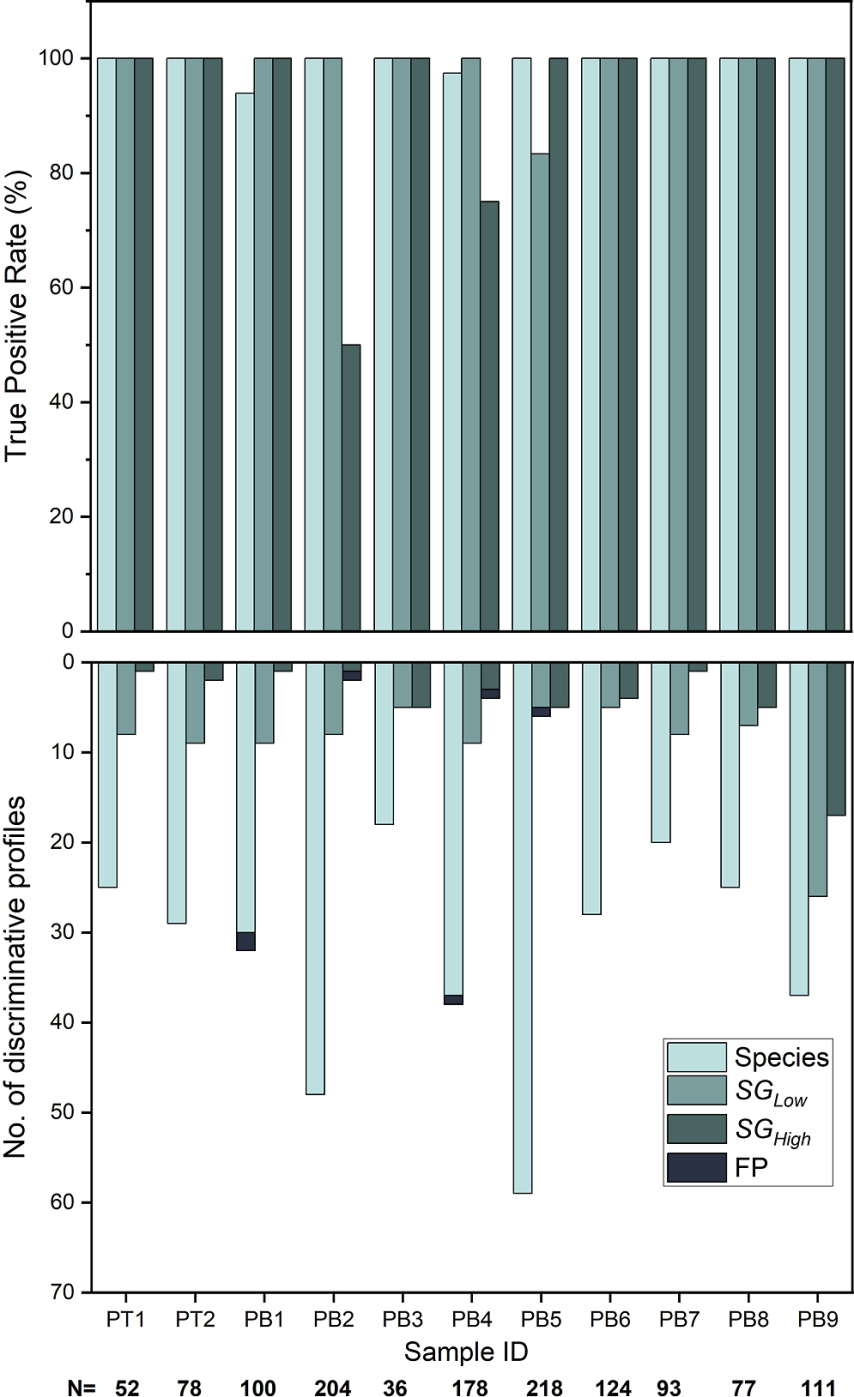
ODM assay can accurately detect *S. pyogenes* isolates at the species and strain-level resolutions. (top) The true positive rate for all 11 isolates at the species and two strain-level resolutions: SG_Low_ and SG_High_. (bottom) The corresponding number of experimental profiles matching discriminatively at each taxonomic resolution, with false positives (i.e., discriminative profiles matching the wrong taxonomic group) marked in dark gray. At the bottom, *N* denotes the total number of DNA fragments imaged for each isolate.

Next, we evaluated the performance of ODM in identifying the *S. pyogenes* samples at the three different *emm* resolutions: *emm* types, which are grouped into *emm* clusters, which in turn are grouped into *emm* patterns. The *emm* typing scheme follows the same algorithm as the strain-level schemes, i.e., all high-confidence matches for an experimental profile must match a single *emm* group for the profile to be considered discriminative at the given *emm* resolution. Only profiles that match discriminatively are reported in the *emm* typing results. There was a strong agreement between the ODM assay and the *emm* typing scheme at all *emm* resolutions: average agreement of 92.2% for *emm* patterns, 91.3% for *emm* clusters, and 85.0% for *emm* types (Figure 4A). For six of the 11 samples, there was a 100% match between the ODM assay and *emm* typing at all *emm* resolutions, while for PB2, PB6, and PB9, most of the fragments matched the correct group at all *emm* resolutions. The agreement between the ODM assay and the *emm* typing was poor for sample PB4. When aligning the core genome of sample PB4 to the core genomes of the reference genomes (Figure 4B), all the closest reference genomes belonged to the type *emm12* (cluster AC-4), while sample PB4 belonged to *emm82* (cluster E3). Thus, the ODM assay identified the *emm* type of this sample as *emm12* and *emm* cluster AC-4, which led to disagreement at all the *emm* resolutions. This is, hence, an example where the *emm* typing scheme fails to describe the true evolutionary relationship between the strains. Furthermore, no result could be obtained for sample PB5 at the *emm* cluster and *emm* type resolutions using the ODM assay because the reference database did not comprise any genomes with the same *emm* type or cluster as sample PB5 (*emm96* belonging to a single protein cluster, Clade Y). Therefore, there were no reference genomes in the database to which the PB5 experimental intensity profiles could correctly match discriminatively. However, the *emm* pattern of sample PB5 could be detected with an agreement rate of 71.4%.

**Figure 4:**
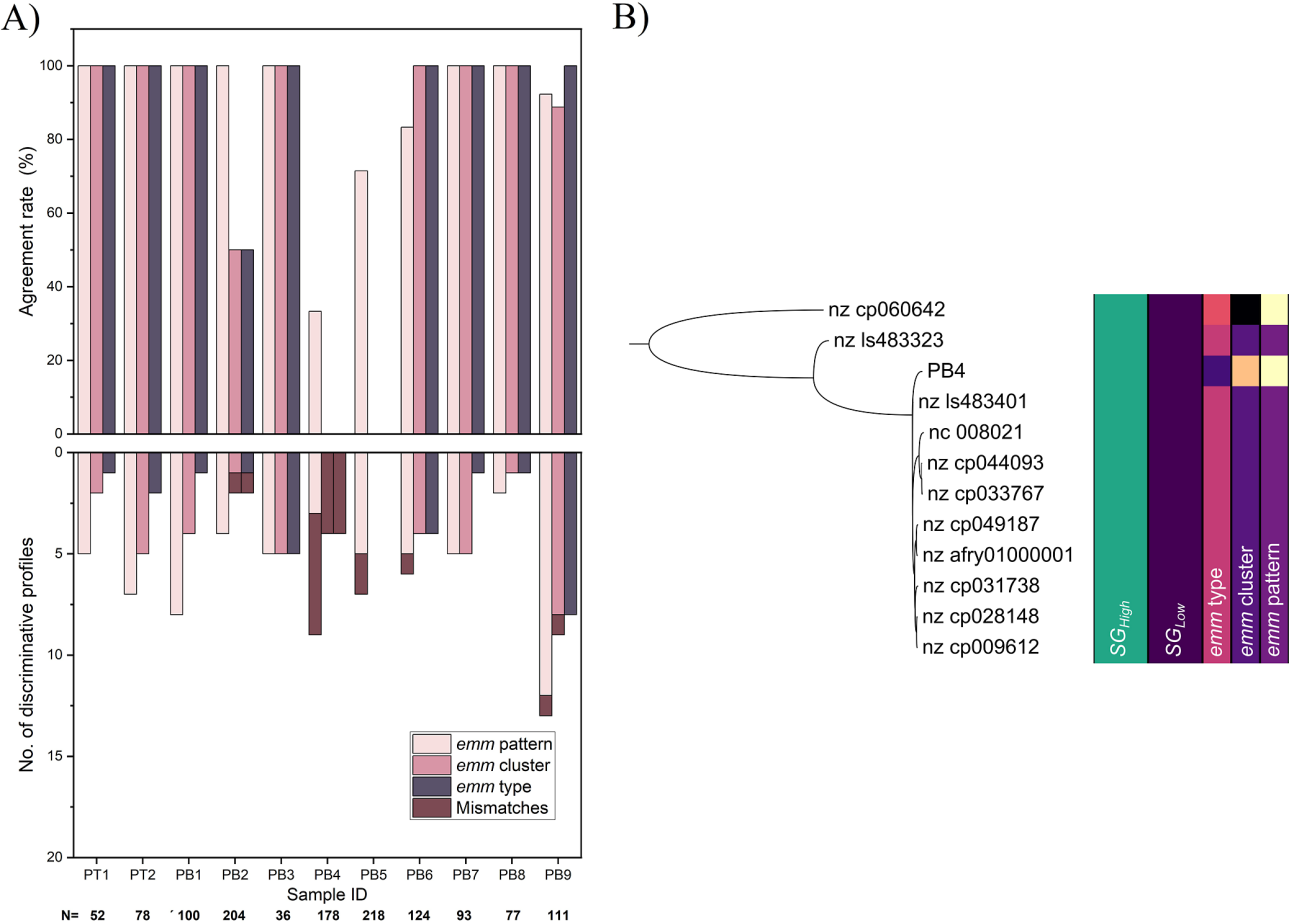
ODM assay can detect *emm* types, *emm* clusters, and *emm* patterns of most of the investigated *S. pyogenes* strains. (A) (top) The agreement rate for the *emm* typing scheme for all 11 isolates at the *emm* pattern (light), *emm* cluster (medium), and *emm* type resolutions (dark). (bottom) The corresponding number of discriminative profiles at each *emm* resolution with mismatches (i.e., discriminative profiles matching the wrong *emm* group) marked in dark plum. *N* denotes the total number of DNA fragments imaged for each isolate. (B) The clade of sample PB4 in the phylogenetic tree, built using all *S. pyogenes* reference genomes and the analyzed samples. All the reference genomes closest related to PB4 belong to *emm12*, while sample PB4 belongs to *emm82*. In our proposed strain group schemes, all members of the clade belong to the same strain group (SG_Low_2 and SG_High_23).

Overall, the proposed ODM-based strain-level typing method performed better when combined with the typing scheme based on the core-genome alignments of the reference genomes compared to the traditional *emm* typing scheme. This is expected since the ODM method utilizes the sequence information on long DNA fragments, albeit at low resolution, while the *emm* typing only uses the exact sequence of a 180 bp long sequence of a single gene. This can be seen in the phylogenetic tree that is based on the core-genome alignment of the reference genomes, where even genomes belonging to the same *emm* pattern (the lowest *emm* resolution) are found on various branches around the tree (Supporting Information, Figure S1). Indeed, the *emm* typing scheme was not developed to reflect the phylogeny of *S. pyogenes* accurately, but to predict virulence based on the M protein. Still, the proposed method was able to identify the correct *emm* type for eight out of 11 isolates, showing that it can provide information on both the strain group and *emm* type of an isolate using the same ODM data.

We argue that our strain group-based typing scheme, derived from a core-genome alignment, could offer a more informative alternative to *emm* typing—not only in terms of virulence profiling but also from an epidemiological perspective. Since the strain groups reflect broader evolutionary relationships among strains, our approach can aid in identifying functional and adaptive differences between the strains, as well as provide high-resolution tracking during outbreak investigations. In contrast, relying on *emm* typing alone might be insufficient if an outbreak is driven by mutations outside the *emm* gene. Moreover, if the *emm* gene undergoes horizontal gene transfer, recombination, etc., the *emm* scheme will lead to spurious results.^17^

Whole-genome sequencing (WGS) and core genome MLST (cg-MLST), the gold standard techniques for strain typing, provide exceptionally high taxonomic resolution up to the clone level. In contrast, the ODM-based assay is constrained by the diffraction limit, offering a resolution of approximately 1000 bp. As a result, genomic mutations smaller than this threshold may go undetected using our approach. Despite this limitation, the ODM-based assay offers several advantages. The ODM-based assay avoids the biases inherent to PCR amplification and sequencing, as it does not rely on primer design or sequencing platforms, offering a more robust and sequencing-independent alternative. Furthermore, the lower information density of the ODM results compared to sequencing-based techniques enables faster alignment and data processing, while also reducing computational and storage requirements. To fully assess the clinical utility of the ODM-based assay, further studies are needed to evaluate its agreement with wg-MLST and *emm* typing for a broader range of clinically relevant strains.

Since the ODM assay can run from start to finish in a few hours, it has the potential to be useful in a clinical context. The assay could be further sped up by reducing the bacterial culture time or entirely forgoing the bacterial culture step. We have previously demonstrated that the ODM assay can accurately type bacteria directly from uncultured urine samples.^12^ However, reliably extracting ultrahigh molecular weight DNA, which is essential for the ODM assay, from complex samples remains a challenge. In this study, we used cultured clinical isolates of *S. pyogenes* extracted from blood and throat secretion samples, which are complex sample matrices. We are currently exploring strategies to improve the extraction of long DNA fragments from clinically relevant sample substrates.

In conclusion, we have demonstrated that ODM, in conjunction with a core-genome alignment-based typing scheme, can be used to successfully identify *S. pyogenes* at the species and strain levels with high accuracy. The proposed technique has a high agreement rate with the *emm* typing scheme and can detect *emm* types, *emm* clusters, and *emm* patterns of most investigated strains. The assay could potentially be deployed in tracking outbreaks of GAS infections and may provide a more robust and comprehensive phylogenetic perspective than the conventional *emm* typing scheme.

## Methods

### Bacterial Strains and Growth Conditions

The bacterial samples were clinical blood and throat isolates from the Department of Laboratory Medicine, Clinical Microbiology at the Karolinska Institutet, Huddinge, Sweden. The bacteria were stored at −80 °C in 10% DMSO (Sigma-Aldrich) and grown in brain heart infusion (BHI) broth (Oxoid), with 1.5% agar for solid medium, at 37 °C. No informed consent was collected from patients, as per the ethical committee assessment (record 2018/273531/2).

### DNA Extraction

The DNA extraction was performed according to a previously described protocol.^11^ Pelleted bacteria, from 300 μL of overnight culture, were resuspended in lysis buffer, mixed with agarose (Sigma-Aldrich) and cast into a plug mold (100 μL). The entire DNA extraction was carried out inside the agarose plug to avoid shearing the DNA. The bacteria were lysed with lysozyme, lysostaphin, and mutanolysin (Sigma-Aldrich) for 2 h at 37 °C, treated with RNase and proteinase K (Sigma-Aldrich) for 1 h at 50 °C, and washed in TE buffer for 1 h at 50 °C. The extracted DNA was released from the agarose plug by melting the agarose in 1× rCutSmart Buffer (New England Biolabs) at 70 °C for 10 min, followed by incubation at 42 °C for 10 min before the addition of 2 μL agarase (Thermo Fisher Scientific, 0.5 U/μL) and a second incubation at 42 °C for at least 1 h. The DNA concentration was determined using a Qubit Fluorometer 2.0 (ThermoFisher Scientific).

### Sequencing

Extraction of total DNA was done using the Illumina Epicenter MasterPure DNA purification kit according to the manufacturer’s instructions. Sequencing libraries were prepared with the Nextera XT Library Prep Kit and sequenced on the Illumina MiSeq platform with a paired-end read length of 300 bp. The sequences were assembled de novo with CLC Genomics Workbench (Qiagen, v21). The CDC’s GAS typing pipeline (Blast-*emm*)^18^ was used to assign *emm* types to the samples (default parameters; reference database downloaded 2023-03-06), and the typing scheme proposed by Sanderson-Smith et al. was used for the annotation of *emm* clusters and *emm* patterns.^6^

### Sample Preparation

The standard competitive binding-based staining protocol described previously was followed for DNA staining.^15^ A known concentration of the isolated sample DNA and λ-DNA (Thermo Scientific) was mixed with YOYO-1 dye (Invitrogen) and netropsin (Sigma-Aldrich) in a 10:1:300 ratio (DNA/YOYO/Netropsin) in 0.5× TBE buffer. The λ-DNA serves as a ruler to determine the extension of the sample DNA in the nanochannel. The mixture was then incubated at 50 °C for 30 min. Following this, the stained sample was diluted 10 times with MQ water to achieve a final buffer concentration of 0.05× TBE, and 2%v/v of 2-Mercaptoethanol (Sigma-Aldrich) was added to minimize oxidative photocleavage.

### Nanofluidic Experiments

The nanofluidic chip used for the experiment consists of 200 straight, parallel nanochannels (100 nm × 150 nm × 500 μm) that are connected to microchannels on either side. The microchannels are also connected to two loading wells on either side. The chips were fabricated using standard nanofabrication techniques, which have been previously described.^13,19^

Each fluorescently stained sample was loaded into the nanofluidic device, and nitrogen gas flow was used to push the DNA molecules into the nanochannels. Molecules were imaged using an epifluorescence microscope (Zeiss Axio Observer Z1), equipped with a 100× oil immersion objective, a Colibri 7 LED light source, a FITC filter set (excitation: BP 475/40, FT 500, emission: BP 530/50), and a Photometrics Prime 95B sCMOS camera. For each experiment, a movie with at least 20 frames was recorded with an exposure of 100 ms.

### Reference Database

A reference database of theoretical profiles was created in the following way. All complete bacterial genome assemblies were downloaded from NCBI RefSeq on 2024-03-12 (the genomes of 38,128 isolates comprising 86,756 sequences). Since plasmids can move between bacterial species and strains, plasmid sequences are not informative in species identification and strain typing. Therefore, all sequences shorter than 500 kbp or containing the word “plasmid” in their FASTA header were removed. Likewise, all sequences with the words “candidatus”, “sp.”, or “bacterium” in their FASTA header were removed, as they are not informative in the species identification. A total of 51,440 sequences were removed, and the remaining 35,316 sequences (from 5488 species) of high-quality assemblies of chromosomal bacterial sequences with a full species name in the FASTA header were converted into the reference database of theoretical profiles as described previously.^12^ See Supporting Information for a list of the accession numbers of all sequences included in the reference database (Table S2).

To understand the evolutionary relationship between the *S. pyogenes* genomes in the database, a phylogenetic tree of the *S. pyogenes* reference sequences was made based on their core genomes. First, all 281 reference sequences of *S. pyogenes* in the reference database were annotated using Prokka^20^ (version 1.14.6; default parameters). From the annotated genomes, the core genomes were identified and aligned using Roary with PRANK (version 3.13.0; parameter-e) and comprised 1136 genes (present in 99% of the genomes). Then, FastTree was used to create a phylogenetic tree from the core genome alignment^21^ (version 2.1.11; parameters-nt-—gtr), which was visualized using iTOL with a midpoint root^22^ (Supporting Information, Figure S1).

The phylogenetic tree was used to assign the *S. pyogenes* references to strain groups (SGs) by trimming the tree at two different branch lengths using R (packages: phytools, version 0.6-99; dendextend version 1.13.4; parameter *h* = 0.015 for SG_Low_ and *h* = 0.010 for SG_High_).^23,24^ When trimming the tree at the short branch length (*h* = 0.010), the 281 reference *S. pyogenes* sequences were divided into 40 SGs with a higher within-group similarity, resulting in an SG scheme of a higher strain-level taxonomic resolution (SG_High_). When the longer branch length was used for the trimming (*h* = 0.015), the references were divided into three SGs with a lower within-group similarity, resulting in an SG scheme of a lower strain-level taxonomic resolution (SG_Low_). All *S. pyogenes* references were also assigned to their *emm* types, *emm* clusters, and *emm* patterns as described above.^18^ See Supporting Information Table S1 for a list of all included *S. pyogenes* reference sequences and their respective assignments to the two analyzed SG schemes, as well as their *emm* types, *emm* clusters, and *emm* patterns.

### Data Analysis

Custom MATLAB and Python scripts were used for the data analysis. The recorded movies were first converted into kymographs using custom MATLAB scripts in the lldev package (10.5281/zenodo.5718208) as previously described.^25^ The kymographs were further aligned and time averaged into average experimental intensity profiles using the HCA package (10.5281/zenodo.5718183). Theoretical profiles corresponding to each sequence in the reference database were generated by calculating the binding probabilities of YOYO-1 and netropsin along the DNA molecules, the point spread function of the imaging system, and the experimental noise, as described earlier.^16,26^ All experimental intensity profiles longer than 150 kbp were aligned to each theoretical profile in the reference database using the HCA package, generating similarity scores based on the Pearson correlation coefficient (PCC), as described previously.^11,12,27^ The matches were then ranked based on their PCC score to identify the best matches.

For each experimental intensity profile, only the high-confidence matches—defined as the highest-scoring database match and any match scoring within the parameter *C*_Diff_ of the highest score—were considered. Hence, *C*_Diff_ was the largest allowed score range for the high-confidence matches. An intensity profile was classified as discriminative if the following criteria were met: (1) all high-confidence matches corresponded to a single taxonomic group (e.g., an SG), and (2) the highest-scoring database match was of sufficient quality (similarity score > parameter *C*_Thresh_). Only discriminative profiles were reported in the typing results, and the profiles with high-confidence matches against multiple taxonomic groups were discarded. The Python code for analyzing the alignment results and identifying intensity profiles that match discriminatively to a single taxonomic group ha been deposited at 10.5281/zenodo.5898280.

There were four parameters affecting the result of the typing analysis: the parameters *L*_Min_ = 250 px and *C*_Thresh_ = 0.5 were set based on previous results.^11,12^ The effect of the parameter *C*_Diff_, i.e. the stringency in the alignment against the reference database, and the taxonomic resolution (species, SG_Low_, SG_High_, *emm* type, *emm* cluster, and *emm* pattern) was investigated using all samples. While varying the parameter *C*_Diff_ (range 0.01–0.08), the effect on the true positive rate (TPR)—i.e. the proportion of discriminative profiles that matched the correct taxonomic group—and the proportion of all experimental profiles that matched discriminatively, was recorded for each sample and then averaged over samples for each taxonomic resolution. For the two analyzed SG schemes, the true SG for each sample was determined by first generating an additional phylogenetic tree based on the 281 *S. pyogenes* reference genomes and all sample genome sequences, using the method described above. The SG of the reference genome closest to each sample genome in the tree was considered the true SG for that sample.

## Supplementary Material





## Data Availability

Raw sequencing data have been submitted to NCBI SRA under BioProject PRJNA1240072.

## Notes

The phylogenetic tree (Figure S1), visualization of the alignment of the theoretical and experimental profiles (Figure S2), and effect of the length rescaling factor (Figure S3) (PDF)
Table S1 (*S. pyogenes* reference genomes) and Table S2 (full list of reference sequences used in the study) (XLSX)
