# Supplementary material for: Strain-Level Typing of Streptococcus pyogenes Using Optical DNA Mapping
Source: ACS Infect Dis. 2025 Oct 15;11(11):3085–92. doi: 10.1021/acsinfecdis.5c00430 (PMC12624843; doi:10.1021/acsinfecdis.5c00430)
Supplement: Supplementary file 1 [file id5c00430_si_001.pdf]

SUPPORTING INFORMATION

Strain-level Typing of *Streptococcus pyogenes* Using Optical DNA Mapping

Radhika N. Kunnath,[1,2#] Zahra Abbaspour,[1#] Anna Johnning,[2,3,4#] Karolin Frykholm,[1,2] Marie Wrande,[5] Albertas Dvirnas,[1,6] Sriram K. K.,[1,2] Christian G. Giske,[7,8] Tobias Ambjörnsson,[6] Linus Sandegren,[5] Erik Kristiansson,[2,3] and Fredrik Westerlund[1,2*]

[1] Department of Life Sciences, Chalmers University of Technology, 412 96 Gothenburg

[2]Centre for Antibiotic Resistance Research in Gothenburg, CARe, Box 440, 40530 Gothenburg, Sweden

[3]Department of Mathematical Sciences, Chalmers University of Technology and the University of Gothenburg 412 96 Gothenburg, Sweden

[4]Department of Systems and Data Analysis, Fraunhofer-Chalmers Centre, 412 88 Gothenburg, Sweden

[5]Department of Medical Biochemistry and Microbiology, Uppsala University, 751 23 Uppsala, Sweden

[6]Centre for Environmental and Climate Science, Lund University, Sölvegatan 12, 223 62 Lund, Sweden

[7]Department of Laboratory Medicine, Karolinska Institutet, 14152 Stockholm, Sweden

[8]Department of Clinical Microbiology, Karolinska University Hospital, 17176 Stockholm, Sweden

[#]These authors contributed equally

[*]E-mail: fredrik.westerlund@chalmers.se

Table of Contents

- Description of additional supporting file
  - Table S1. Table with information about the *S. pyogenes* reference genomes
  - Table S2. List of NCBI accession numbers of all reference sequences used in the study
- Fig S1. Phylogenetic tree of *S. pyogenes* reference genomes
- Fig S2. Visualization of the alignment of the experimental and theoretical profiles
- Fig S3. Effect of the length rescaling factor on the results

Description of Additional Supporting Files

Tables S1 S2.xlsx with the following sheets:

Table S1. A table of the 281 *Streptococcus pyogenes* reference genomes with their respective NCBI RefSeq accession number (accno), strain type (ST), *emm* pattern, *emm* cluster, *emm* type, and strain group in the higher (SG_High) and lower (SG_Low) strain-level typing schemes.

Table S2. A list of the NCBI accession numbers of all reference sequences used to generate the reference database.

Phylogenetic tree of *S. pyogenes* reference genomes

Figure on the following page.

Figure S1. Phylogenetic tree of the 281 *Streptococcus pyogenes* reference genomes, based on their core-genome alignment. The leaf labels correspond to the NCBI RefSeq accession number. The two innermost colored circles indicate which strain group each reference genome belongs to in two schemes at the strain-level resolution: SG_{High} and SG_{Low}. The three outermost colored circles indicate which *emm* type, *emm* cluster, and *emm* pattern each reference genome belongs to, respectively, with white being “Unknown”.

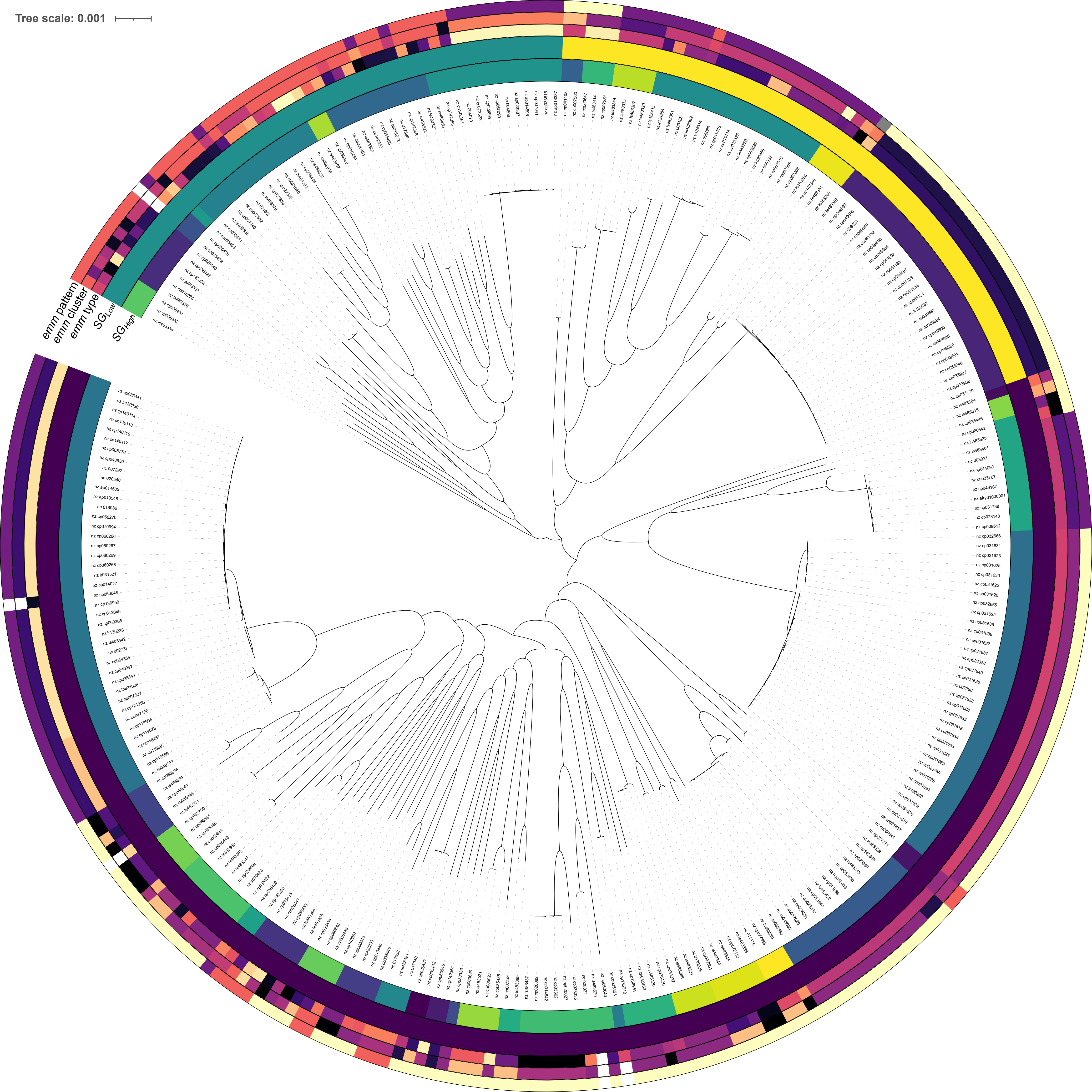
Tree scale: 0.001
emm pattern
emm cluster
emm type
SG_Low
SG_High

Visualization of the alignment of experimental and theoretical profiles

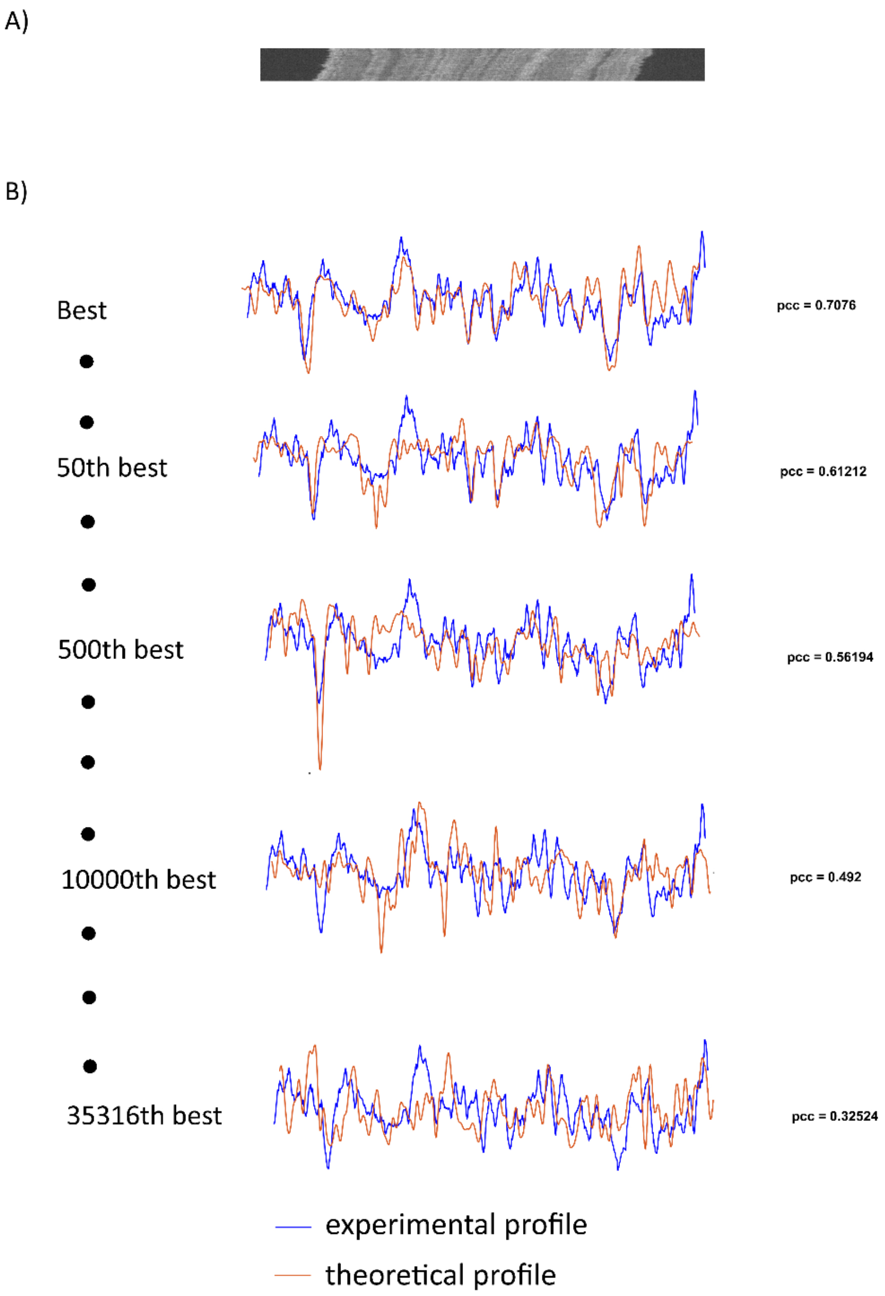

Figure S2. Visualization of the alignment process. **A)** A kymograph obtained from imaging a DNA molecule from sample PT1. **B)** The experimental intensity profile (blue) corresponding to the kymograph in A) is matched to all the 35,316 theoretical profiles in the database (red). The similarity between the experimental and theoretical profiles is evaluated in terms of the Pearson correlation coefficient score (pcc). The matches are ranked in decreasing order of similarity, and all matches with a score within C_{Diff} of the best match are defined as high-confidence matches. When all high-confidence matches are assigned to a single taxonomic group, the experimental profile is considered to match discriminatively to that taxonomic group.

Effect of the length-rescaling factor

For each experiment, the nanometer-to-base pair ratio (nm/bp) was estimated from the average length of imaged λ-DNA molecules of known length (48,500 bp). In the data analysis, the theoretical profiles are first length-rescaled to attain the same extension as that of the examined experimental profile. To account for the molecule-to-molecule fluctuations in extension, the experimental intensity profiles are rescaled along the length axis within a user-defined range (the upper value of that range is referred to as the maximum rescaling factor). For each molecule, all the length-rescaled experimental profiles are compared to the theoretical profiles and the maximum Pearson correlation coefficient-based score obtained during this comparison is used to determine the similarity between the theoretical and the experimental profiles. Figure S2 shows the effect of the maximum length-rescaling factor on the proportion of discriminative profiles and the true positive rate (TPR) of the method at the species level. The experimental profiles from all eleven samples were pooled together to study the effect of the parameter C_{Diff} and the maximum length-rescaling factor on the performance of the method. In this study, the maximum length-rescaling factor was varied between 0% and 10% in steps of 2.5%. At a C_{Diff} of 0.05, the TPR is greater than 99% for maximum length-rescaling factors from 5% to 10%. The proportion of discriminative matches decreases with increasing C_{Diff} and increases with the maximum length-rescaling factor. Thus, a maximum length-rescaling factor of 10% and a C_{Diff} of 0.05 were chosen for the analysis as they maximized the number of discriminative profiles while ensuring a TPR of greater than 99%.

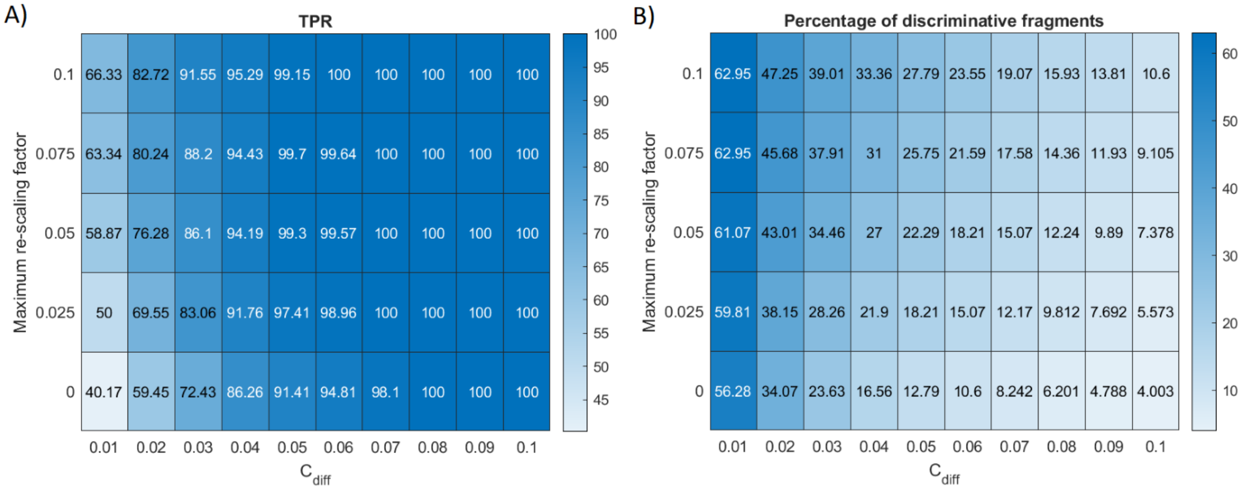

Figure S3. Effect of the maximum length-rescaling of the experimental intensity profiles on **A)** the true positive rate (TPR) and **B)** the proportion of discriminative profiles at the species level with varying database matching stringency (parameter C_{Diff}).
